# Prevalence and sociodemographic determinants of tobacco use among adults in Pakistan: findings of a nationwide survey conducted in 2012

**DOI:** 10.1186/1478-7954-11-16

**Published:** 2013-09-03

**Authors:** Sara Ijaz Gilani, David A Leon

**Affiliations:** 1 Gallup Pakistan, Islamabad, Pakistan; 2 London School of Hygiene and Tropical Medicine, London, United Kingdom

**Keywords:** Tobacco, Cigarettes, Prevalence, Sociodemographic determinants

## Abstract

**Background:**

Smoking is one of the leading causes of preventable mortality. The World Health Organization recommends that countries should monitor tobacco use regularly. In Pakistan, the last national study on smoking in the general population was conducted in 2002 to 2003.

**Methods:**

We conducted a cross-sectional survey of a nationally representative sample of men and women living in rural and urban areas of four main provinces of Pakistan from March through April 2012. Face-to-face in-house interviews were undertaken using a pre-tested structured questionnaire that asked about smoking and other forms of tobacco use. Multistage stratified random area probability sampling was used. To determine the national prevalence of tobacco use, the sample was weighted to correspond to rural–urban population proportions in each of the four provinces as in the 1998 census conducted by Pakistan’s Population Census Organization. Associations between sociodemographic variables and tobacco use were investigated using multivariable robust regression.

**Results:**

Out of 2,644 respondents (1,354 men and 1,290 women), 354 men and 4 women reported being current cigarette smokers. The weighted prevalence of current cigarette smoking was 15.2% (95% confidence interval [CI]; 11.2, 19.3) overall, 26.6% (95% CI: 19.1, 34.1) among males, and 0.4% (95% CI: -0.2, 1.0) among females. Among females, 1.8% (95% CI: 0.4, 3.1) used any smoked tobacco and 4.6% (95% CI: 1.8, 7.4) used any smokeless tobacco daily or on some days of the week. Among males, odds of current cigarette smoking decreased with increasing level of education (OR = 0.75; 95% CI: 0.68, 0.84) and increased with having a father who used tobacco (OR = 2.11; 95% CI: 1.39, 3.22) after adjusting for other sociodemographic characteristics. Lower household income was associated with current cigarette smoking among rural males only (odds ratio [OR] = 0.67; 95% CI: 0.48, 0.92 per category increase in monthly household income).

**Conclusion:**

A large proportion of males smoked cigarettes. Cigarette use was negligible among females, but they used other forms of tobacco. Low education was a determinant of cigarette smoking among males irrespective of socioeconomic status and area of residence. Tobacco control campaigns should target uneducated and rural poor men and monitor all forms of tobacco used by the population.

## Background

Smoking is one of the leading causes of preventable morbidity and mortality [1]. According to the World Health Organization (WHO), there are nearly 1 billion smokers in the world. Smoking kills 6 million people every year. These include mostly current smokers and ex-smokers (5 million approximately). Each year, however, nearly 600,000 people die of hazards of secondhand smoke as well [2].

Two thirds of the world’s smokers live in low- and middle-income countries [2]. This rising burden of smoking in these countries is attributed to aggressive marketing strategies of large multinational tobacco companies [3]. Smoking leads to premature death and is a huge economic burden on health systems and families of smokers, especially in developing countries with already limited resources [2].

In order to control the tobacco epidemic and protect the health of world citizens, WHO recommends six evidence-based measures called MPOWER, which are: monitoring of tobacco use and prevention strategies, protection of people from tobacco smoke, offering help to smokers for quitting, warning public about adverse effects of smoking, enforcing bans on tobacco advertisement and promotion, and raising taxes on tobacco products [1]. Monitoring of tobacco use is an important strategy so that countries can keep track of tobacco burden as well as gauge the effect of preventive strategies. According to one estimate, however, only 59 countries of the world conduct adult tobacco surveys regularly once every five years [2].

Pakistan is the sixth most populous country of the world with a population of nearly 190 million [4]. About one-third of the population lives in urban areas. There is considerable migration from rural to urban areas and it is estimated that, by 2030, half of the people in Pakistan will be living in urban areas [5]. About 22.3% of the population live below the poverty line. The expenditure on health is low i.e. 2.6% of gross domestic product [4]. Pakistan faces a “double burden” of disease with communicable as well as non-communicable diseases (NCD) causing morbidity and mortality. According to an estimate, nearly 54.9% of the deaths in the country are caused by NCDs [6]. Tobacco use is one of the main preventable risk factors for NCDs. Pakistan signed the Framework Convention for Tobacco Control (FCTC) in 2004 [7].

In Pakistan, a number of ad hoc studies have been conducted on prevalence and determinants of smoking, but these have limited geographic coverage and have focused on selected segments of society [8-14]. The figures usually quoted for national prevalence of tobacco use are either from the National Health Survey of Pakistan (1990–1994) or from the World Health Survey (2002–2003) [15,16]. According to the report of the World Health Survey carried out in Pakistan, the prevalence of any form of smoked tobacco among adults was 19.9% (33.5% for males and 6.2% for females) [16]. Almost a decade has passed since this survey, but no more recent data has been published. Though conventional cigarettes are the main form of tobacco consumed globally, tobacco is also used in other forms such as hookah, naswar, tobacco in paan and gutka in Pakistan [17,18]. WHO recommends that all forms of tobacco use must be monitored, but no data for prevalence of smokeless tobacco was reported from Pakistan in the 2011 WHO Report (based on World Health Survey 2002–2003 data) [7]. Pakistan was not present in the current wave of Global Adult Tobacco Survey (GATS), which reported smoking prevalence from 14 low- and middle-income countries, although it is scheduled to participate in Wave 3 [19]. Given this paucity of recent data, we conducted a study to measure the prevalence of self-reported cigarette smoking and other forms of tobacco use among adults, and to explore the socio-demographic determinants that are associated with current smoking among adults in Pakistan in 2012.

## Methods

The study was based on a cross-sectional survey conducted with a nationally representative sample of adults (≥ 18 years of age) living in any of the four main provinces. The population of these provinces constitutes 95% of the total population of the country.

### Sampling

The sample of the study was selected by multi-stage stratified random area probability sampling. The universe for the sample was adult (≥ 18 years of age) men and women living in households of four provinces of Pakistan: Punjab, Sindh, Khyber Pakhtunkhwa, and Balochistan. People living in Gilgit Baltistan, Federally Administered Tribal Areas, Azad Jammu, and Kashmir, institutions, military areas, and the homeless population were excluded. The excluded population constituted less than 5% of the total population [20].

Two-stage stratification into province and rural–urban strata was used. Within each stratum, the primary sampling unit (PSU) was selected from a sampling frame consisting of urban census circles or mauzas/revenue villages (for urban and rural strata respectively). The PSU was selected using a probability-proportionate-to-size method. The probability of selection of PSU was proportionate to its population size, as estimated by the 2012 population projections of 1998 census, which was the latest census data available in the country at the time of the study. Thus the larger census circles and mauzas had relatively greater probability of being selected in the sample. Each PSU was divided into four to six segments, and then one of the segments was randomly selected. The interviewer was instructed to go to the center of the segment, select a random starting house on a random lane, and then go to every third household following the right-hand rule. The target respondent in the selected household was adult male or female of 18 years or older who consented to be interviewed for the study. If there was more than one eligible respondent in a household, one was randomly selected using the lottery method. At each PSU, ten respondents were interviewed. The sample was equally divided among males and females.

A sample of approximately 2,500 respondents was decided. This gives an error margin of ± 2% to 3% at the 95% CI (assuming the proportion of smokers to be 20% as reported in World Health Survey 2002–2003) [16,21].

### Questionnaire

A module of questions on tobacco use was developed using previously validated questionnaires [15,22]. This module was then administered as part of an omnibus survey regularly conducted by Gallup Pakistan, a leading survey research company in Pakistan. Gallup Pakistan has a panel of adult household men and women across the country and these respondents are interviewed in person weekly by trained interviewers using structured questionnaires. This omnibus survey explores public opinion on various issues such as society, lifestyles, consumer behavior, health, education, and politics. Current cigarette smokers were defined as those who reported smoking at the time of interview. Past smokers were defined as those who had smoked at least 100 cigarettes or five packs in their entire lives, but were not currently smoking [15,23]. Questions on use of other forms of tobacco used in Pakistan were also included [17,18,23-25].

The determinants of smoking, such as gender, age, education, and area of residence, which have been reported in literature, were included [15,16,26]. In order to measure the socioeconomic status of respondents, questions from the World Bank’s Poverty Score Card were used [27,28]. The score card has ten simple questions about members of household and household assets (such as electronic goods, automobiles, livestock, and agricultural land). The responses to individual questions are given specific scores and are added to give a total poverty score for the respondent [27]. A lower score indicates more poverty. Questions on migration and family history of tobacco use were also included.

The questionnaire was developed and administered in Urdu, which is the national language of the country and is widely understood. However, the interviewers were provided with translations of some terms in other local languages (e.g., Pushto and Sindhi) to be used if required during field work.

### Field work

Face-to-face, in-house interviews were conducted with the randomly selected respondents using pre-tested questionnaires by a trained team of about 400 interviewers led by field supervisors of Gallup Pakistan. At least 10% of the field work of each interviewer was verified by back-checking with a supervisor revisiting the household to check the authenticity of the interview conducted by the interviewer. The field work was carried out from March to April 2012.

### Data processing

All of the data from questionnaires was double-entered into SPSS and checks were made to identify discrepancies that were then corrected.

### Ethical considerations

The study was approved by Ethical Review Committee of the London School of Hygiene and Tropical Medicine (Application No.011/12). Gallup Pakistan is a registered survey company and is authorized to conduct public opinion surveys, so no formal ethical approval was needed from any institution in Pakistan. Interviewers were trained to introduce themselves and seek verbal informed consent from the respondents. Written consent was not considered due to the high illiteracy level in the country and the simple nature of the survey [4]. Confidentiality and anonymity of respondents was maintained. The personal details (e.g., names, addresses) were not stored in the analytical dataset; instead, each respondent was given a unique identification number. Datasets were password protected and only accessible to research staff. Field work and data processing was carried out by Gallup Pakistan according to the code of conduct laid out by the European Society for Opinion and Marketing Research [29].

### Statistical analysis

The dataset was analyzed using Stata Version 11 [30]. Frequency/percentages were calculated for categorical variables. For continuous variables, summary measures of mean and median were calculated. To estimate national prevalence figures, the sample data was weighted to correspond to the rural–urban proportion for each of the four provinces, as in the Census 1998 [20]. This was done for descriptive analysis only. Analysis of determinants of smoking was done on unweighted data as it gives associations of smoking with sociodemographic variables for the sample only [31].

To look for association between sociodemographic variables and self-reported smoking, chi-squared tests were performed at the 5% significance level. Odds ratios with 95% CI were also calculated for current smoking as the outcome. For ordered categorical variables like age, a score test for trend was also performed.

Age was considered an a priori confounder and all estimates for any association were adjusted for age of the respondent. Considering the difference in socioeconomic conditions across urban and rural areas, analysis was carried out to see any interaction by area of residence with other determinants of smoking [32,33].

All multivariable analyses were performed by robust regression. Robust regression accounted for clustering within the same PSU/cluster. A series of regression models were constructed in a structured fashion [34]. Age was considered an a priori confounder and included in the baseline model. The variable with the smallest p value in bivariable analysis, education (p = 0.001), was selected first and robust logistic regression of current smoking was performed with age and education and each other variable (i.e., income, poverty score, location, parents’ tobacco use) in turn. The likelihood ratio test, using a 5% significance level, was used to see if variables improve the fit of model or not. Next, variables such as father’s tobacco use, province, and socioeconomic status were added sequentially and the above steps repeated until no variable had p < 0.05. The models were built for all males (national sample) and then separately for urban and rural males, respectively.

Missing data due to non-response to individual questions was not a major problem. Multivariable regression analyses were restricted to respondents for whom data on all concerned variables was available.

## Results

### Sample profile

The survey was conducted with 2,644 respondents. The characteristics of the unweighted sample, that was predominantly urban, are detailed in Table 1. There were almost equal numbers of men and women. The mean unweighted age was 34 ± 10 years (Range: 18–90 years), and the weighted mean age was 35 years (95% CI: 33, 36). The monthly household income was not reported by nearly 12% of the respondents. Among those who reported the income, mean household income was 18,027.4 ± 12,154.8 Rupees (Rs.) per month in the unweighted data. Upon weighting, average monthly household income was 14,177.3 Rs. (95% CI: 12,316.7, 16,038.0). Poverty score ranged from 12 to 122. The mean unweighted poverty score was 59.5 ± 19.7, and when weighted it was 57.9 (95% CI: 54.6, 61.3).

**Table 1:** Sociodemographic characteristics of survey respondents (unweighted profile)

**Characteristics**	**n**	**%**
**1. Gender**		
Male	1354	51.2
Female	1290	48.8
**2. Age (years)**		
18–20	151	5.7
21–30	971	37.0
31–40	818	30.9
41–50	450	17.0
51–60	118	4.7
>60	32	1.2
Missing	98	3.7
**3. Education**		
Illiterate	211	8.0
No formal education but can read	107	4.1
Up to Primary (≤ 5 years)	255	9.6
Up to Middle (6–8 years)	376	14.2
Up to Matric (9–10 years)	633	23.9
Up to Intermediate (11–12 years)	411	15.5
Graduate/Post graduate/Professional Education	605	23.3
Missing	46	1.7
**6. Monthly household income (Rs.)**^**1**^		
< 3000	62	2.3
3001–7000	308	11.7
7001–10,000	364	13.8
10,001–15,000	530	20.1
15,001–30,000	707	26.7
>30,000	337	12.8
Missing	336	12.7
**7. Poverty score**		
Up to 20 (Poorest)	11	0.4
21–40	300	11.4
41–60	1336	50.5
61–80	639	24.2
81–100	176	6.7
>100 (Richest)	182	6.9
**8. Location**		
Rural	353	13.4
Urban	2291	86.6
**9. Province**		
Punjab	1414	53.5
Sindh	835	31.6
Khyber Pakhtunkhwa	181	6.9
Balochistan	214	8.1
**Total**	**2644**	**100**

Note: ^1^Pakistani rupee (Rs.) = approximately 0.01 US dollar (US$).

### Prevalence of tobacco use

Out of 2,644 respondents, 354 men and 4 women reported being current cigarette smokers. The weighted prevalence of different forms of tobacco used is shown in Table 2. The current cigarette smoking was 15.2% overall, 26.6% among males and 0.4% among females. The total tobacco prevalence (smoked or smokeless) was 34.9% among males and 5.1% among females. Weighted prevalence for use of specific smoked and smokeless tobacco products among men and women is shown in Table 3. Use of other tobacco products was lower than cigarette use among males. However, other tobacco products were used more by women than cigarettes.

**Table 2:** **Prevalence of all forms of tobacco use**^1^**(weighted**^**2**^**)**

**Form of tobacco**	**Male**		**Female**		**All**	
	**n**	**% (95% CI)**	**n**	**% (95% CI)**	**n**	**% (95% CI)**
Cigarettes^3^	354	26.6 (19.1, 34.1)	4	0.4 (−0.2, 1.0)	358	15.2 (11.2, 19.3)
Any smoked^3,4^	374	31.4 (23.0, 39.6)	46	1.8 (0.4, 3.1)	420	18.2 (14.0, 22.5)
Any smokeless^3,5^	233	15.1 (7.1, 23.2)	82	4.6 (1.8, 7.4)	315	10.5 (6.0, 15.0)
Any tobacco use^6^	463	34.9 (26.4, 43.5)	88	5.1 (2.2, 8.0)	551	21.7 (17.4, 26.1)

Notes:

^1^Users used product “daily or on some days of the week”. Individuals might be using multiple forms of tobacco but are counted once only.

^2^The sample data is weighted such that the urban–rural proportion in each of the four provinces is the same as that reported in the 1998 Population and Housing Census. The weighted percentage gives a weighted national average.

^3^They might be using other forms as well.

^4^Cigarettes/bidis/hookah/shisha.

^5^Naswar/tobacco in paan/gutka.

^6^Any smoked/smokeless tobacco.

**Table 3:** **Use of non-cigarette tobacco products among the sample (weighted**^1^**)**

**Tobacco product**	**Users**^**2**^					
	**Male**		**Female**		**All**	
	**n**	**% (95% CI)**	**n**	**% (95% CI)**	**n**	**% (95% CI)**
Hookah^3^	121	13.8 (5.8, 21.8)	39	1.3 (0.2, 2.4)	160	8.3 (3.8, 12.7)
Sheesha^3^	102	8.3 (0.8, 15.8)	41	1.3 (0.2, 2.5)	143	5.2 (1.0, 9.4)
Bidi^4^	104	8.8 (1.2, 16.4)	40	1.3 (0.2, 2.4)	144	5.5 (1.2, 9.7)
Naswar^5^	157	11.1 (3.4, 18.9)	44	2.2 (0.2, 4.3)	201	7.2 (2.9, 11.5)
Gutka^6^	142	9.4 (1.9, 16.9)	50	2.7 (0.5, 5.0)	192	6.4 (2.1, 10.8)
Tobacco in paan^7^	160	11.5 (3.8, 19.3)	63	2.2 (1.0, 3.5)	223	7.4 (3.1, 11.7)

Notes:

^1^The sample data is weighted such that the urban–rural proportion in each of the four provinces is the same as that reported in the 1998 Population and Housing Census. The weighted percentage gives a weighted national average.

^2^Users used product “daily or on some days of the week”.

^3^“Flavored tobacco is burned in a smoking bowl covered with foil and coal. The smoke is cooled by filtration through a basin of water and consumed through a hose and mouth-piece.” (17, page 26).

^4^“Hand-rolled Indian cigarette; temburni leaf rolled into a conical shape together with flaked tobacco and secured with a thread.” (24, page 56).

^5^“Naswar is a mixture of sun-dried, sometimes only partially cured, powdered local tobacco (N.rustica), ash, oil, flavouring agents (e.g. cardamom, menthol) colouring agents (indigo) and in some areas, slaked lime.” (18, page 52).

^6^“Sun-dried, roasted, finely chopped tobacco, areca nut, slaked lime and catechu mixed together with several other ingredients such as flavourings and sweeteners.” (18, page 50).

^7^“It is also called Betel quid with tobacco and has four main ingredients; betel quid, areca nut, slaked lime and tobacco. Various tobacco preparations are used in un-processed, processed or manufactured forms.” (18, page 49).

There was some variation among the use of specific tobacco products by province. Among men and women combined, Hookah was used more in Punjab (12.4%) compared to Sindh (2.1%), Khyber Pakhtunkhwa (4.1%), and Balochistan (0.5%). Naswar was more prevalent in Khyber Pakhtunkhwa (10.6%) than Punjab (8.6%), Sindh (3.3%), and Balochistan (0.9%).

The proportion of past cigarette smokers among males was 1.8% (95% CI: 0.4, 3.2). More than half of the male smokers (56.2%; 95% CI: 44.4, 67.9) expressed the desire to quit smoking. On average, current male smokers smoked 13 (95% CI: 5.9, 20.2) cigarettes each day. The majority of them (68%; 95% CI: 55.6, 80.3) smoked up to a half of a pack of cigarettes daily, and 84.9% (95% CI: 76.1, 93.7) started smoking before the age of 25 years.

There was substantial agreement among both smokers as well as non-smokers that smoking is harmful for health. On the whole, 87.9% (95% CI: 83.2, 92.6) of men and women said that smoking is very harmful for health, 9.2% (95% CI: 5.6, 12.8) said that it was somewhat harmful, and 1.5% (95% CI: -0.4, 3.3) said that smoking was not harmful. The remaining 1.5% (95% CI: 0.6, 2.3) gave no response.

### Sociodemographic determinants of cigarette smoking among males

Because of the small number of current cigarette smokers among females, we have restricted the analysis on determinants of smoking to male respondents only. Table 4 shows age-adjusted ORs for current cigarette smoking for men among different socioeconomic strata. Taking urban and rural males together, there was an association between higher age and odds of current smoking. Smoking was more prevalent among those with lower education. Current smoking was lower in Khyber Pakhtunkhwa than Punjab province. Men whose fathers used some tobacco had higher odds of current smoking. Area of residence, mothers’ tobacco use, and migration did not have significant association with male cigarette use.

**Table 4:** **Association of socio-demographic characteristics with current cigarette smoking among male respondents stratified by area of residence**^1^

**Characteristics**	**Age-adjusted OR (95% CI)**		
	**All Pakistan**	**Urban**	**Rural**
**Age (years)**			
≤ 30	1 [Ref]^2^	1 [Ref]	1 [Ref]
31-50	1.48 (1.10, 1.98)	1.56 (1.13, 2.15)	0.99 (0.48, 2.03)
>50	1.60 (0.97, 2.64)	1.73 (1.03, 2.93)	0.91 (0.19, 4.35)
OR per change in category (95% CI)	1.34 (1.08, 1.67)	1.40 (1.11, 1.77)	0.97 (0.51, 1.82)
**2. Education**			
Illiterate/No formal education	1 [Ref]	1 [Ref]	1 [Ref]
Up to primary (≤ 5 years)	0.71 (0.39, 1.30)	0.84 (0.45, 1.57)	0.70 (0.22, 2.24)
Up to middle (6–8 years)	0.61 (0.34, 1.10)	0.74 (0.36, 1.51)	0.57 (0.22, 1.50)
Up to matric (9–10 years)	0.40 (0.22, 0.71)	0.56 (0.29, 1.08)	0.18 (0.06, 0.50)
Up to intermediate (11–12 years)	0.30 (0.17, 0.54)	0.40 (0.21, 0.75)	0.13 (0.02, 0.70)
Graduate/post graduate/professional education	0.26 (0.15, 0.47)	0.35 (0.18, 0.67)	0.12 (0.03, 0.50)
OR per change in category (95% CI)	0.79 (0.71, 0.86)	0.82 (0.74, 0.90)	0.68 (0.55, 0.83)
**3. Poverty Score**			
Bottom quartile (Poorest)	1 [Ref]	1 [Ref]	1 [Ref]
Second quartile	1.16 (0.83, 1.62)	1.22 (0.85, 1.76)	0.99 (0.46, 2.12)
Third quartile	0.92 (0.61, 1.38)	0.99 (0.64, 1.52)	0.59 (0.21, 1.66)
Top quartile (Richest)	1.04 (0.70, 1.56)	1.07 (0.71, 1.61)	1.07 (0.30, 3.73)
OR per change in category (95% CI)	0.99 (0.87, 1.13)	1.00 (0.88, 1.14)	0.98 (0.66, 1.45)
**4. Monthly household income (Rs.)**^**3**^			
< 3000	1 [Ref]	1 [Ref]	1 [Ref]
3001–7000	0.74 (0.33, 1.68)	0.83 (0.26, 2.68)	0.63 (0.20, 1.99)
7001–10,000	0.56 (0.24, 1.32)	0.63 (0.18, 2.12)	0.65 (0.19, 2.26)
10,001–15,000	0.51 (0.20, 1.33)	0.81 (0.24, 2.68)	0.06 (0.01, 0.39)
15,001–30,000	0.52 (0.21, 1.26)	0.73 (0.24, 2.25)	0.18 (0.03, 1.05)
>30,000	0.60 (0.24, 1.54)	0.83 (0.26, 2.67)	0.17 (0.02, 1.79)
OR per change in category (95% CI)	0.93 (0.80, 1.07)	1.00 (0.87, 1.16)	0.57 (0.41, 0.80)
**5. Fathers’ tobacco use**^**4**^			
Yes	2.30 (1.61, 3.29)	2.33 (1.58, 3.44)	2.13 (0.84, 5.40)
No	1 [Ref]	1 [Ref]	1 [Ref]
**6. Mothers’ tobacco use**^**4**^			
Yes	1.16 (0.64, 2.08)	1.15 (0.61, 2.18)	1.48 (0.34, 6.38)
No	1 [Ref]	1 [Ref]	1 [Ref]
**7. Province**			
Punjab	1 [Ref]	1 [Ref]	1 [Ref]
Sindh	0.86 (0.55, 1.35)	0.89 (0.55, 1.43)	0.84 (0.24, 2.96)
Khyber Pakhtunkhwa	0.34 (0.20, 0.58)	0.34 (0.20, 0.58)	0.29 (0.07, 1.24)
Balochistan	0.91 (0.51, 1.60)	1.11 (0.66, 1.86)	-
**8. Location**			
Urban	1 [Ref]	NA^5^	NA
Rural	1.21 (0.67, 2.15)	NA	NA
**9. Migration from village to city (urban residents only)**			
Yes	NA	1.32 (0.92, 1.88)	NA
No	NA	1 [Ref]	NA

Notes:

^1^Analysis restricted to respondents with no missing data for each explanatory variable and age. Analysis is by robust regression.

^2^[Ref] is Reference category for OR,

^3^*Rs*. Pakistani Rupee.

^4^The question was on any form of tobacco use by mother or father.

^5^*NA* not applicable.

When stratifying by urban/rural area of residence, we see that low education was associated with higher smoking among both urban and rural males, but the effect was stronger among rural males with a marginally significant p value for test for interaction (p = 0.042). Monthly household income showed no systematic association with current smoking among urban males, but for rural males, OR for current cigarette smoking decreased with increasing household income (p value of test for interaction = 0.001). Age and fathers’ tobacco use showed association with current cigarette smoking among urban men but there was no evidence of interaction by area of residence for these associations.

The results of our investigation of the independent effect of each of the sociodemographic variables on self-reported current cigarette smoking among males are shown in Table 5. In the sample as a whole, and among urban and rural men examined separately, the odds of current smoking decreased with increasing level of education and were higher for men whose father had used tobacco. Khyber Pakhtunkhwa had significantly lower odds of current smoking among men than Punjab province. Other characteristics did not show significant associations with cigarette use in the fully adjusted model. When stratified by urban–rural location, there was a strong association between low household income and current cigarette smoking among rural men though this association was not significant for urban men. Higher age only showed a strong association with cigarette smoking among urban men.

**Table 5:** Age and fully adjusted odds ratios for current cigarette smoking among males by sociodemographic characteristics

**All males (N = 1025)**		
**Characteristics**	**Age-adjusted OR**	**Fully adjusted OR**
	** (95% CI)**	** (95% CI)**^**1**^
**Age** (OR per category)^2^	1.18 (1.01, 1.36)	1.15 (0.98, 1.34)
**Education** (OR per category)^3^	0.77 (0.69, 0.85)	0.75 (0.68, 0.84)
**Monthly household income** (OR per category)^4^	0.92 (0.80, 1.07)	1.04 (0.90, 1.21)
**Poverty score** (OR per quartile from poorest to richest)	1.03 (0.90, 1.19)	1.13 (0.97, 1.32)
**Province**		
Sindh vs. Punjab	0.79 (0.49, 1.28)	0.92 (0.57, 1.50)
Khyber Pakhtunkhwa vs. Punjab	0.26 (0.14, 0.46)	0.30 (0.18, 0.50)
Balochistan vs. Punjab	0.66 (0.30, 1.48)	0.90 (0.45, 1.81)
**Location** (Rural vs. Urban)	1.12 (0.54, 2.28)	0.87 (0.46, 1.64)
Those with **fathers who used tobacco** vs. those with fathers who do not use tobacco	2.06 (1.38, 3.09)	2.11 (1.39, 3.22)
Those with **mothers who used tobacco** vs. those with mothers who do not use tobacco	1.11 (0.58, 2.12)	0.87 (0.41, 1.84)
**Urban males (N = 853)**		
**Age** (OR per category)^2^	1.22 (1.03, 1.43)	1.21 (1.03, 1.43)
**Education** (OR per category)^3^	0.81 (0.72, 0.90)	0.80 (0.71, 0.89)
**Monthly household income** (OR per category)^4^	0.99 (0.83, 1.14)	1.08 (0.92, 1.27)
**Poverty score** (OR per quartile from poorest to richest)	1.05 (0.91, 1.22)	1.13 (0.97, 1.32)
**Province**		
Sindh vs. Punjab	0.81 (0.49, 1.35)	0.92 (0.55, 1.55)
Khyber Pakhtunkhwa vs. Punjab	0.27 (0.14, 0.50)	0.31 (0.18, 0.52)
Balochistan vs. Punjab	1.01 (0.71, 1.42)	1.21 (0.84, 1.74)
**Migration from village to city**^**5**^	1.30 (0.91, 1.87)	1.29 (0.88, 1.89)
Those with **fathers who used tobacco** vs. those with fathers who do not use tobacco	2.03 (1.32, 3.12)	2.10 (1.35, 3.29)
Those with **mothers who used tobacco** vs. those with mothers who do not use tobacco	1.13 (0.58, 2.21)	0.84 (0.39, 1.84)
**Rural Males (N = 148)**		
**Age** (OR per category)^2^	0.82 (0.49, 1.38)	0.77 (0.40, 1.47)
**Education** (OR per category)^3^	0.60 (0.48, 0.77)	0.63 (0.50, 0.81)
**Monthly household income** (OR per category)^4^	0.59 (0.42, 0.82)	0.67 (0.48, 0.92)
**Poverty score** (OR per quartile from poorest to richest)	0.90 (0.54, 1.48)	1.15 (0.75, 1.76)
**Province**		
Sindh vs. Punjab	0.68 (0.16, 2.84)	0.90 (0.28, 2.83)
Khyber Pakhtunkhwa vs. Punjab	0.17 (0.04, 0.82)	0.35 (0.05, 2.36)
Those with **fathers who used tobacco** vs. those with fathers who do not use tobacco	2.08 (0.66, 6.53)	3.07 (1.04, 9.02)
Those with **mothers who used tobacco** vs. those with mothers who do not use tobacco	1.83 (0.36, 9.26)	4.41 (0.77, 25.20)

Notes:

^1^For all males: adjusted for age, education, province and fathers’ tobacco use. For Urban Males; adjusted for age, education, province and fathers’ tobacco use. For rural males: adjusted for age, education, fathers’ tobacco use, and monthly household income.

^2^Age group categories: 18–20, 21–30, 31–40, 41–50, 51–60, and >60 years, respectively.

^3^Education categories: illiterate/no formal education, up to primary (≤ 5 years), up to middle (6–8 years), up to matric (9–10 years), up to intermediate (11–12 years), and graduate/post-graduate/professional education, respectively.

^4^Monthly household income categories: <3000, 3001–7000, 7001–10,000, 10,001-15,000, 15,001-30,000 and >30,000 Rupees respectively.

^5^OR for migrants vs. non-migrants.

## Discussion

### Prevalence of tobacco use

This national household survey of adults in Pakistan showed self-reported prevalence of current cigarette smoking to be 15%. Among females, it was negligible, whereas 31% of males smoked any form of tobacco (cigarette, bidi, or hookah). This includes 27% of males who smoked cigarettes only, whereas 4% additionally smoked other forms of tobacco at the time of the survey. Comparison with previous studies confirms the persistence of smoking prevalence among males. In the World Health Survey (2002–2003), 32.4% of males were using any smoked tobacco [7,16]. Cigarettes were the main form of tobacco used. This is in contrast to neighboring countries like Bangladesh and India and where bidi also has a major share [35-37].

The persistence of smoked tobacco use among males over the last decade is a serious cause for concern for two reasons. First, the absolute number of male smokers at risk of various health problems has increased tremendously due to the population increase over these years. This is evident from the increase in cigarette consumption in the country [38]. Second, the prevalence of cigarette smoking has not declined, which is in contrast to the trend seen in most developed countries [17]. This raises serious questions on the effectiveness of tobacco control program in Pakistan. Though Pakistan has signed the Framework Convention of Tobacco Control, there are issues with implementation of tobacco control laws. According to the 2011 WHO Report on the Global Tobacco Epidemic, Pakistan scores very poorly in terms of compliance with smoke-free legislation for public places. Also, there were no direct bans on advertising and promotion of tobacco in electronic media (television, radio, newspapers) nor on billboards or at points of sale. Services for helping smokers to quit smoking are also limited [7]. This is also evident from a very low cessation rate (1.8%) among males in the present study.

The current study showed lower prevalence of smoked tobacco among females than previous surveys. 6.2% reported use of any smoked tobacco in World Health Survey (2002–2003) in Pakistan, whereas in the present study, 1.8% reported to be using any smoked tobacco [7,16]. This difference might be due to underreporting in the current study. However, our estimates are similar to the proportion of females who smoke tobacco in India (2.9%; 95% CI: 2.6, 3.4) and Bangladesh (1.5%; 95% CI: 1.1, 2.1) as reported in Global Adult Tobacco Survey (2008–2010) [19]. Another explanation for the discordance between our findings and those of earlier surveys in Pakistan is that there may have been overestimation of use of smoked tobacco among females in previous surveys. Alternatively, there may have been a real decrease in use among females in Pakistan over the last decade, which in our view is unlikely. However, it should be noted that direct comparison with the previous surveys is problematic as they asked about any smoked tobacco (e.g., cigarette, bidi, or hookah) in one combined question, whereas we explored each category of use separately.

The use of other forms of tobacco among females included more than cigarettes in the present study. Almost 5% females reported using smokeless tobacco. This might be because of greater cultural acceptability of these tobacco products rather than cigarettes among females in this region [39]. A similar pattern was seen in a study in India where smoking prevalence among females was negligible, whereas other smokeless tobacco use was considerable [37]. Females in Pakistan might be still relatively protected from the cigarette epidemic, but other culturally acceptable forms of tobacco are more prevalent. Considering the prevalence of these forms of tobacco among females, it is a cause for concern that there is no regular monitoring of smokeless tobacco in the country, nor are there any health warnings on smokeless tobacco products [7].

Globally, smoking rates are lower among females than males, especially among developing countries where female smoking is 9% compared to 22% for females in developed countries. Women in the developing world, however, have been targeted by tobacco advertising as they represent an untapped market for the industry [3]. This has increased smoking prevalence among females in some developing countries [40,41]. It is feared that smoking prevalence will increase further among females in the developing countries and converge with male prevalence as previously seen in many developed countries. This will have a huge public health impact [17,42].

### Determinants of cigarette smoking

There was a strong negative linear trend with increasing education level that was evident across all socioeconomic strata as well as urban and rural areas. This association between smoking and lack of education has been reported in other studies in Pakistan, Bangladesh, and India [11,35-37,43]. This might be due to lack of awareness about the effects of tobacco products [11,35,36].

Smoking was higher in rural males with low household income compared to those with higher incomes. This association with poverty has been seen in other studies [35,36,44]. Why poor people smoke is attributed to lack of awareness about adverse effects of smoking or the stresses of poverty causing individuals to take up smoking as a coping mechanism [35,36]. It is interesting to note that, when adjusted for education level, there was no significant association between income and smoking. Thus, the association with income appears to be secondary to confounding with education and lack of awareness among the poor rural population, or it might be related to cultural differences across socio-economic groups. Nevertheless, as rural poor men smoke more, an increase in tobacco taxes might reduce tobacco consumption; according to Mushtaq et al., a 10% increase in cigarette prices could decrease consumption by 11.7% [38]. The decrease might be greater among the poor who are considered to be more responsive to the increase in price [41].

The present study showed some association of current smoking with increasing age. A similar trend was seen in the World Health Survey (2002–2003) in Pakistan where prevalence of smokers among those younger than 30 years was 10% and among those aged 60 to 69 was approximately 30% [16]. Similarly, higher prevalence in individuals older than 45 years was seen in studies in Bangladesh and India [35,45]. Lower prevalence in younger ages might suggest a shift in taking up smoking toward higher ages. A study in India also suggested that smoking starts at a later age than in Europe and North America [46], but it could also be due to underreporting by younger- respondents. A qualitative study of Pakistanis in the United Kingdom showed that smoking was considered more acceptable for older men compared to younger individuals [47].

Studies have looked at the adverse health effects of passive smoking, especially for young children in households where adults smoke [48]. Also, children are prone to poor health and malnutrition when their fathers spend their limited income on buying cigarettes [3]. However, the present study showed how tobacco use by fathers could increase uptake of cigarette smoking among male children even after adjustment for sociodemographic factors. A previous study on adolescents also found that boys who had a smoker in the family were more likely to smoke themselves [49].

Smoking was considerably lower among males in Khyber Pakhtunkhwa compared to the Punjab province. This might be due to higher use of naswar in Khyber Pakhtunkhwa as noted in the present study and elsewhere [43]. Rural poor and rural uneducated men had the highest odds of current smoking when compared to their urban counterparts. This suggests a potential target group for future tobacco control campaigns.

### Limitations of the study

Prevalence of smoking was based on self-reporting by the respondents and not by biomarker measurement (e.g., serum cotinine levels) [50]. There may be underreporting as respondents might consider smoking to be socially undesirable (e.g., among females). The questionnaire was administered by interviewers and thus there was risk of interviewer bias. We attempted to minimize these issues by using previously validated questions, pretesting of the questionnaires, interviewer training and supervision, as well as back-checking.

Though multistage random sampling was used for selection of respondents, it could be subject to some selection bias if the interviewer did not follow sampling instructions properly. The survey was limited to household population in four provinces and findings cannot be generalized to segments that were excluded as discussed in methods section. The sampling frame was based on 2012 projections of the 1998 census. Population of some areas might have changed during this period, but it was the latest available census data in the country at the time of the survey.

Non-response to most questions was less than 5%, which is acceptable for such surveys, but for some variables (e.g., income), data was missing in about 12% of cases. This could affect the results if smoking differed among those who did not report their income.

The findings were adjusted for some confounders like age, but there could be residual confounding especially if there was an error in measuring the variables (e.g., poverty score, income, age, etc.).

The sample size of approximately 2,600 gave an error margin of ± 2% to 3% at 95% CI. The analysis on males only was performed on a sample of 1,000 to 1,200 with an error margin of ± 3% to 4%. Smaller segment analysis (e.g., rural males) had a greater sampling error and thus some associations might have been missed.

The study was a cross-sectional study and the determinants and prevalence of smoking were measured at one point in time. Therefore, it is not possible to determine the direction of causal relationships between smoking and some of the factors like income.

### Recommendations for tobacco control program in Pakistan

These findings reinforce the need for strengthening the Tobacco Control Program in Pakistan. Specifically, we suggest the following:

•Because no significant decrease in cigarette use among males was seen in Pakistan over the last decade, the Tobacco Control Program needs to review its strategies, which do not appear to be having an impact. Though legislation exists that bans tobacco use in public places, it is not being implemented. There is no ban on direct promotion or advertisement of tobacco products in electronic media. Under such circumstances, simply putting health warnings on cigarette packaging is not enough to control the tobacco epidemic in the country. More than half of male smokers express the desire to quit, but the proportion of past smokers (quitters) is less than 2% so efforts should be made to support smokers in quitting.

•Female cigarette smoking is at a low level at the current time, and continued efforts are needed to keep it at low levels. Efforts to decrease use of other tobacco products by females are also needed.

•The rural poor and rural uneducated are at high risk of smoking so tobacco control campaigns should tailor their messages to this socially disadvantaged and vulnerable group. This might require modifications of the message, its language, and mode of delivery. An increase in taxation on tobacco products can decrease its consumption, especially by the poor. Investing in education would have additional benefits for tobacco control.

•Tobacco control campaigns need to devise messages to deter youth who have seen their parents using tobacco from taking up smoking themselves.

•Regular surveys on prevalence and determinants of all forms of tobacco use in the general population should be carried out at least every five years. Unless we keep track of the progress made by the Tobacco Control Program, we will be unable to control this epidemic.

### Future research

A larger study that allows analysis at the provincial level and has a larger rural sample to give estimates with reasonable error margins is required. Moreover, pathways through which education protects against smoking and poverty leads to increased smoking need to be studied, especially in the rural context.

## Conclusion

A considerable proportion of adult males in Pakistan reported themselves as current cigarette smokers. Cigarette use was negligible among females, though use of other tobacco products was higher. No other form of tobacco was as widely used as conventional cigarettes. Lower education, province of residence, and having a father who used tobacco were the main risk factors for current cigarette smoking among males. In addition, low income was a risk factor for rural males.

## Abbreviations

CI: Confidence interval; FCTC: Framework convention for tobacco control; GDP: Gross domestic product; NCD: Non-communicable diseases; OR: Odds ratio; PSU: Primary sampling unit; Rs.: Pakistani Rupee; SPSS: Statistical package for the social sciences; WHO: World health organization

## Competing interests

The authors declare that they have no competing interests.

## Authors’ contributions

SIG led the study including the design of the survey, undertook the data analysis, and drafted the manuscript. DAL advised at all stages of the study, including data analysis and interpretation, and contributed to the drafting of the manuscript. Both authors read and approved the final manuscript.
